# Protection Against Rabbit Hemorrhagic Disease Virus Strains: Efficacy of a New Commercial Recombinant RHDV2 Capsid Protein VP60 Vaccine

**DOI:** 10.3390/vaccines14020123

**Published:** 2026-01-27

**Authors:** Mireia Fontseca, Eva Perozo, Guillem Nadal, Sandra Gascón-Torrens, Jordi Montané, Ricard March, Marta Sitjà

**Affiliations:** HIPRA S.A., Avinguda La Selva, 135, 17170 Amer, Spain; mireia.fontseca@hipra.com (M.F.); eva.perozo@hipra.com (E.P.); guillem.nadal@hipra.com (G.N.); jordi.montane@hipra.com (J.M.); ricard.march@hipra.com (R.M.); marta.sitja@hipra.com (M.S.)

**Keywords:** rabbit hemorrhagic disease, vaccine efficacy, onset of immunity, duration of immunity, virus-like particles

## Abstract

**Background/Objectives**: Rabbit hemorrhagic disease (RHD) is an incurable, highly contagious disease caused by different RHD virus (RHDV) strains, such as the coexisting RHDV2 and classical RHDV. Disease control is based on vaccination using different or polyvalent vaccines. This study assessed the protective efficacy, onset of immunity (OOI), and duration of immunity (DOI) of a new recombinant vaccine containing a single active substance developed to target both strains: against RDHV2, highly virulent RDHV2 (hvRHDV2), and classical RDHV strains. **Methods**: This study included six randomized, controlled, blinded trials in clinically healthy New Zealand White rabbits. Rabbits were grouped to receive the recombinant vaccine or placebo (1:1 ratio) and challenged with RHDV2 (2^15^ hemagglutination [HA], infective dose; *n* = 39 for duration and *n* = 43 for onset), hvRHDV2 (2^15^ HA; *n* = 48 and *n* = 40), or classical RHDV (2^12^ hemagglutination [HA], infective dose; *n* = 20 and *n* = 22) strains to evaluate OOI and DOI. **Results**: Rabbits receiving the vaccine showed a lower mortality than those receiving placebo upon challenge with any of the three strains. OOI trials showed that vaccinated rabbits exhibited higher levels of antibodies against RHDV2 than controls seven days post-vaccination. DOI trials revealed that, compared with controls, vaccinated rabbits had increased levels of antibodies against RHDV2 across all time points assessed, at least until the day of the viral challenge with any of the RHDV strains (approximately 12 months post-vaccination). **Conclusions**: This recombinant vaccine is the first to show a durable and robust protection against all tested strains, including the RHDV2, hvRHDV2, and classical RHDV strains, underscoring its potential as a comprehensive tool for RHD prevention.

## 1. Introduction

Rabbit hemorrhagic disease (RHD) is an infectious, highly contagious disease that causes acute hepatitis and disseminated intravascular coagulation in pet, farming, and wild European rabbits (*Oryctolagus cuniculus*). It was first detected in 1984 in China but quickly spread to other countries worldwide, becoming endemic in many of them. In addition, RHD has a negative ecological impact on populations of wild rabbits and their predators [1].

The causal agent of RHD is the RHD virus (RHDV), which belongs to the genus *Lagovirus* of the family *Caliciviridae.* RHDV is a non-enveloped, single-stranded RNA virus with a small-sized capsid (35–40 nm diameter) [1]. This capsid protects the RNA and consists of 180 subunits of a single structural protein named VP60 [1,2]. Until 2010, six genotypes of RHDV had been identified (G1–G6) [3]. In 2010, another strain, phylogenetically and antigenically different from the classical RHDV GI.1 (hereafter classical RHDV), was identified and named RHDV GI.2 (hereafter RHDV2) [4].

Both strains still coexist, although RHDV2 has become the predominant one and is associated with most current RHD cases worldwide [4]. The classical RHDV entails a high mortality rate (80–90%) and affects rabbits of any age. However, in animals younger than 6–8 weeks, the infection is subclinical. In contrast, RHDV2 has a highly variable mortality rate (50–80%) depending on the strain, but clinical manifestations and deaths occur at a younger age (from 7 days old onwards) [3]. In addition, the pathogenicity of the RHDV2 strain has increased over time and the original strain currently coexists with the highly virulent RHDV GI.2 (hereafter hvRHDV2) strain [5].

At present, there is no cure available for RHD. Thus, RHD control is based on disease prevention strategies like vaccination. However, vaccines against classical RHDV showed limited protection against RHDV2 [6,7,8]. Consequently, and given the emergent and critical situation in the rabbit sector due to the rapid spread of the disease, vaccines against RHDV2 were developed shortly after RHDV2 identification. Nevertheless, these first vaccines against RHDV2, which consisted of inactivated viruses obtained from the liver of infected animals, conferred limited protection against the classical RHDV strain. Therefore, rabbits had to be vaccinated with different or polyvalent vaccines, resulting in higher costs for breeders [9].

Considering the urgent need for protection against the hvRHDV2 strain, a new method has been developed to produce a recombinant vaccine with a single active substance that confers protection against RHDV2 and classical RHDV strains. This study aimed to assess the protective efficacy, onset of immunity (OOI), and duration of immunity (DOI) of this new recombinant vaccine against different RHDV strains corresponding to RDHV2, hvRDHV2, and classical RDHV.

## 2. Materials and Methods

### 2.1. Study Animals and Experimental Design

This work consisted of six randomized, controlled, blinded trials including a total of 227 clinically healthy New Zealand White rabbits sourced from a certified Spanish breeder farm. Animals received the test vaccine or placebo and were subsequently challenged with different RHDV strains (i.e., RHDV2, hvRHDV2, and classical RHDV) to evaluate vaccine efficacy in terms of onset of immunity (OOI) and duration of immunity (DOI). In DOI trials, all the animals were vaccinated against myxomatosis (MIXOHIPRA-H, HIPRA, Amer, Spain) one month before vaccination.

To the extent possible, the trials were designed following the requirements described in the European Pharmacopoeia—specifically in the monographs “Rabbit hemorrhagic Disease Vaccine (Inactivated)” [10], which only refers to classical RHDV, and “Evaluation of Efficacy of Veterinary Vaccines and Immunosera” [11]—and those described in the EMEA note for guidance “Duration of protection achieved by veterinary vaccines” (EMEA/CVMP/682/99-final).

Rabbits were required to be free from antibodies against RHDV2 and classical RHDV, and to be of the minimum age recommended for vaccination (i.e., 30 days) to be included in the trial. However, young rabbits are not susceptible—at least not fully—to classical RHDV infections [12]. Thus, a challenge at a young age is not advisable. In order to exclude natural immunity against classical RHDV in young rabbits, and following the Ph. Eur. [10], challenges with classical RHDV were performed in 10-week-old rabbits.

This study was conducted based on the Good Laboratory Practices described in Spanish Royal Decree 1369/2000, of 19 July 2000, and the OECD Principles of Good Laboratory Practice (99/11/CE), as revised in 1997. The study protocol was reviewed and approved by the Animal Experimentation Commission of the Government of Catalonia (authorization code 10740), which acts as the competent Institutional Animal Care and Use Committee, in accordance with Directive 2010/63/EU and Spanish Royal Decree 53/2013.

In each trial, animals were randomly distributed at a 1:1 ratio to receive the test vaccine (vaccine group) or placebo (control group). A single 0.5 mL dose of the vaccine or sterile phosphate-buffered saline was administered to vaccine or control groups, respectively, by subcutaneous injection in the interscapular area, using a sterile syringe connected to a sterile 25G × 5/8” needle. To ensure blind administration, the treatment dispenser (the person in charge of treatment administration and not involved in data collection) was the only person aware of the correspondence between the products administered and the study groups.

In OOI trials, animals were challenged 7 days after the administration of the vaccine or placebo, whereas in DOI trials, the challenge was performed 12 months later (Table 1). The challenges were performed intramuscularly with a single dose of one of the following RHDV strains:

- RHDV2: strain V-1037, isolated from an RHD outbreak in Spain in 2013.
- hvRHDV2: strain V-1171, isolated from an RHD outbreak in France in 2020, and associated with a higher mortality than strain V-1037 [13].
- Classical RHDV: strain V-4764, isolated from an RHD outbreak in Spain in 1997, administered 14 days after vaccination or placebo.

In all cases, the animals were euthanized 14 days after being challenged, by intracardiac administration of sodium pentobarbital (Dolethal^®^, 1 mL/kg) following anesthesia with a combination of xylazine, zolazepam, and tiletamine administered intramuscularly (0.5 mL/kg; corresponding to 10 mg/kg of each component at a concentration of 20 mg/mL).

**Figure 1:**
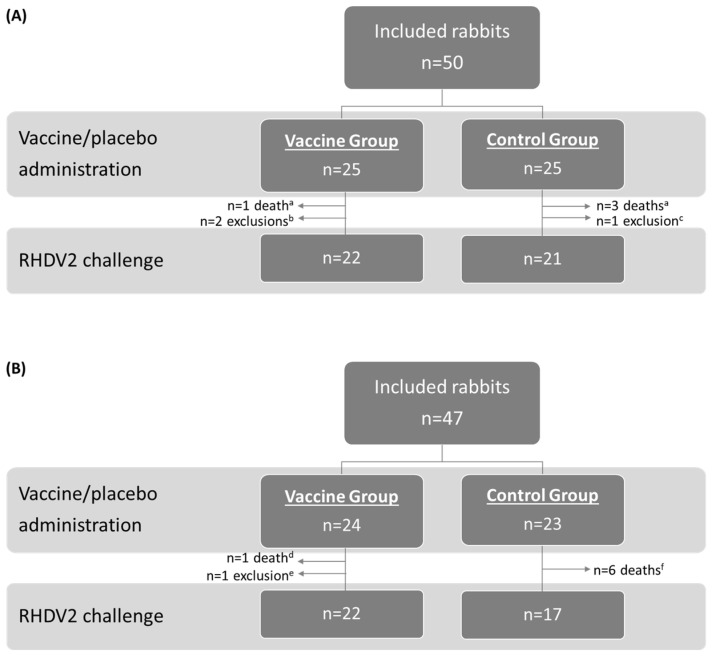
Study population and allocation into treatment groups in studies evaluating onset of immunity (**A**) and duration of immunity (**B**) of the vaccine against an RHDV2 strain. ^a^ Caused by coccidiosis. ^b^ Due to not proper vaccination and lack of seroconversion (*n* = 1) or digestive problems (*n* = 1). ^c^ Due to depression. ^d^ Caused by colibacillosis. ^e^ Due to paralysis of the hindquarters. ^f^ Caused by colibacillosis (*n* = 2), nephropathy (*n* = 1), or unknown reasons (*n* = 3). RHDV2, rabbit hemorrhagic disease virus type 2.

**Figure 2:**
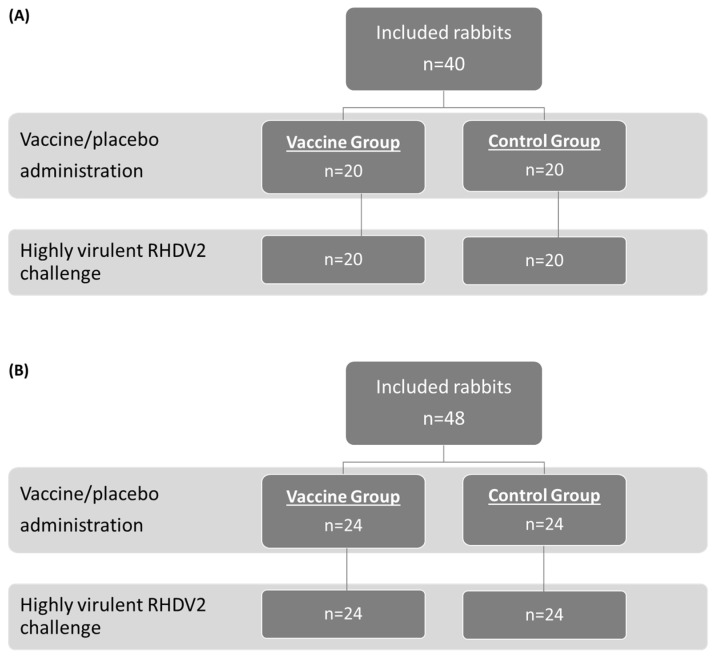
Study population and allocation into treatment groups in studies evaluating onset of immunity (**A**) and duration of immunity (**B**) of the vaccine against a highly virulent RHDV2 strain. RHDV2, rabbit hemorrhagic disease virus type 2.

**Figure 3:**
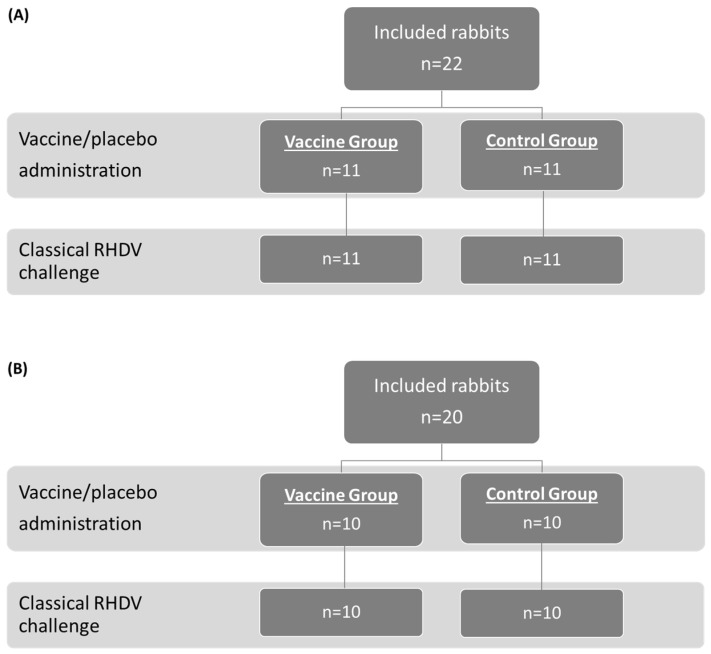
Study population and allocation into treatment groups in studies evaluating onset of immunity (**A**) and duration of immunity (**B**) of the vaccine against a classical RHDV (GI.1) strain. RHDV, rabbit hemorrhagic disease virus.

The study population and allocation into treatment groups for the OOI (A) and DOI (B) trials are shown in Figure 1 (RHDV2), Figure 2 (hvRHDV2), and Figure 3 (classical RHDV).

### 2.2. Commercial Vaccine

The test vaccine, Yurvac^®^ RHD (HIPRA, Amer, Spain), consists of the recombinant VP60 capsid protein derived from a single RHDV2 field strain, which auto-assembles into virus-like particles (VLPs), as the active substance, and light mineral oil as the adjuvant, forming an oil-in-water emulsion. The antigen VP60 was obtained through recombinant DNA technology (yeast host-vector system). The adjuvant used for this vaccine has three main properties: it allows for gradual and continuous release of the antigens; it provides a vehicle capable of transporting the emulsified antigen through the lymphatic system to distant sites, creating additional foci of antibody formation; and it interacts with mononuclear cells, such as phagocytic cells and antigen presenting cells. As specified in the summary of product characteristics, the vaccine must be stored between 2 °C and 8 °C but must reach room temperature before administration.

### 2.3. Variables and Assessments

The serological status and response were evaluated through the levels of antibodies against RHDV2 at different time points: (1) the day of vaccination, before administering the vaccine or placebo; (2) approximately every 42 days after vaccination until the challenge (only in trials determining the DOI); (3) the day of the challenge, before performing it; and (4) 14 days after the challenge, before euthanasia. The levels of antibodies against RHDV2 were assessed by obtaining 1 mL of blood samples from the auricular vein and performing the hemagglutination inhibition assay (HAI) using an RHDV2 antigen heterologous to the vaccine component. This antigen was obtained by collecting and macerating livers from rabbits previously inoculated with RHDV2, inactivating the virus with a 10% volume of 0.1 M binary ethyleneimine (BEI) at 36–38 °C for 7 h and neutralizing residual BEI with 20% sodium thiosulphate. Seropositivity for RDHV2 was defined as log_2_ HAI/50 μL ≥ 3.0, which was the quantification limit established during the validation of this assay.

In addition, liver samples were obtained from all animals dying within the 14-day period after the challenge to assess the presence of RHDV2, hvRHDV2, and classical RHDV as appropriate, using the hemagglutination technique [3].

Moreover, clinical signs in the animals of depression, body condition, dyspnea, nasal discharge, ocular discharge, and other symptoms (e.g., diarrhea, respiratory signs) (Table S1) were also recorded daily throughout the trials.

### 2.4. Outcomes

The OOI and DOI of the vaccine were assessed based on mortality, clinical signs, and serological response within the 14 days after the challenge in vaccinated and control groups.

The primary outcome was the mortality caused by the strain used in the challenge, defined as the proportion of animals that died per group with the presence of RHDV2, hvRHDV2, or classical RHDV strains, as appropriate, in the liver. The secondary outcomes were the presence of clinical signs characteristic of RHD (twice-daily monitored by the same trained personnel) and the serological response from placebo/vaccine administration to the end of the 14-day period following the challenge.

The efficacy of the vaccine against classical RHDV was defined as per the Ph. Eur. [10], i.e., ≥80% deaths with typical signs of RHD in the control group at 120 h after the challenge and ≥90% survival in the vaccine group, showing no signs of RHD [10]. In the case of RHDV2 strains, the efficacy of the vaccine was defined as statistically significant differences in mortality rates between control and vaccine groups in favor of the latter.

### 2.5. Statistical Analyses

Considering the mortality found in a previous study using the same hvRHDV2 strain for the challenge [14], the number of animals per group in trials using RHDV2 strains was established as 20. In contrast, according to Ph. Eur. requirements [10], the number of animals per group in trials using the classical RHDV for the challenges was ten.

Quantitative variables were described as the mean and standard deviation (SD), whereas categorical variables were expressed as frequencies and percentages. Comparisons between vaccinated and control groups were conducted using the Mann–Whitney U test for quantitative variables and the Chi-squared test for categorical variables.

The statistical threshold was established at a two-sided α level of 0.05, and all analyses were performed with Microsoft^®^ Excel 2013 (Microsoft Corp., Redmond, WA, USA), SPSS (version 22), or R Studio (version 4.3.3).

## 3. Results

### 3.1. Protection Against RHDV2

#### 3.1.1. Mortality and Clinical Signs

The OOI trial showed that, after the challenge with RHDV2, 0 (0.0%) rabbits died in the vaccine group and 18 (85.7%) in the control group, with most of these deaths (*n* = 16, 88.9%) occurring between 24 and 48 h after the challenge. The analysis of liver samples showed the presence of RHDV2 in most rabbits dying after the challenge (*n* = 14, 77.8%).

In the DOI trial, zero (0.0%) and nine (52.9%) rabbits died in the vaccine and control groups, respectively, with all deaths occurring within 24 and 72 h after the challenge. The analysis of liver samples showed the presence of RHDV2 in all rabbits dying after the challenge.

**Figure 4:**
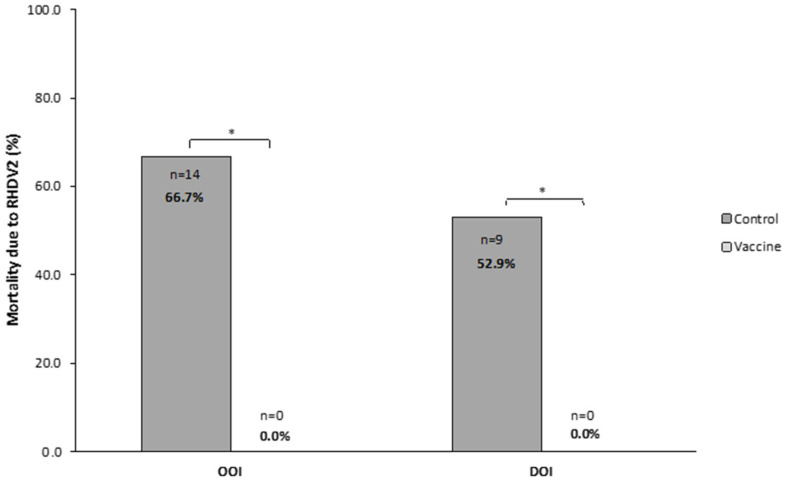
Mortality caused by rabbit hemorrhagic disease virus type 2 (RHDV2) in vaccine and control groups of studies evaluating the onset of immunity (OOI) and duration of immunity (DOI) against an RHDV2 strain. * *p* < 0.05.

Therefore, OOI and DOI trials showed that the mortality due to RHDV2 was statistically significantly higher (*p* < 0.05) in rabbits receiving the placebo than in those receiving the vaccine (Figure 4).

Regarding clinical signs, none of the rabbits from any treatment group showed clinical signs related to RHD within 14 days after the challenge.

#### 3.1.2. Serological Response

**Figure 5:**
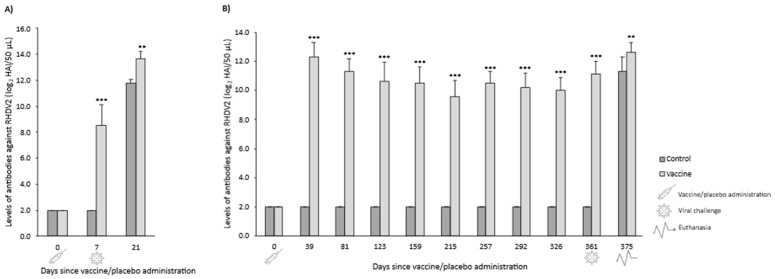
Mean (SD) levels of antibodies against rabbit hemorrhagic disease virus type 2 (RHDV2) in vaccine and control groups of studies evaluating the onset of immunity (**A**) and duration of immunity (**B**) against an RHDV2 strain. ** *p* < 0.01; *** *p* < 0.001 vs. controls.

In both OOI and DOI trials, control rabbits were seronegative for RHDV2 from the day they received the placebo (day 0) until the day they were challenged (day 7 for OOI trial and day 361 for DOI trial). The levels of antibodies against RHDV2 were statistically significantly higher (*p* < 0.01) in vaccinated rabbits compared with control rabbits at all time points assessed after administering the vaccine or placebo, respectively (Figure 5).

### 3.2. Protection Against Highly Virulent RHDV2

#### 3.2.1. Mortality and Clinical Signs

In the OOI trial, 0 (0.0%) rabbits died in the vaccine group and 19 (95.0%) in the control group, with all deaths occurring within 72 h after the challenge. The analysis of liver samples showed the presence of the hvRHDV2 strain in all rabbits dying after the challenge.

In the DOI trial, 3 (12.5%) and 22 (91.7%) rabbits died in the vaccine and control groups, respectively, with all deaths in the control group occurring within 24 and 120 h after the challenge. All rabbits dying after the challenge presented the hvRHDV2 strain in their liver.

**Figure 6:**
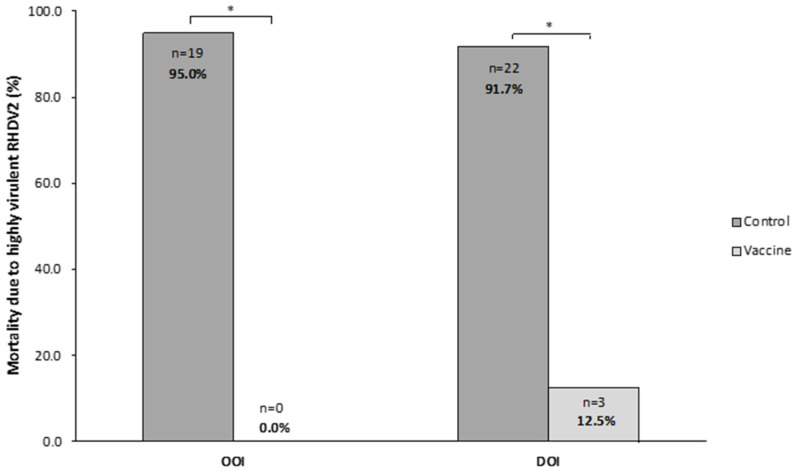
Mortality caused by a highly virulent rabbit hemorrhagic disease virus type 2 (hvRHDV2) variant in vaccine and control groups of studies evaluating onset of immunity (OOI) and duration of immunity (DOI) against a highly virulent RHDV2 strain. * *p* < 0.05.

Thus, in both trials, mortality due to the hvRHDV2 strain was statistically significantly higher (*p* < 0.05) in rabbits receiving the placebo than in those receiving the vaccine (Figure 6).

With respect to clinical signs, none of the rabbits from any treatment group showed clinical signs related to RHD within 14 days after the challenge.

#### 3.2.2. Serological Response

**Figure 7:**
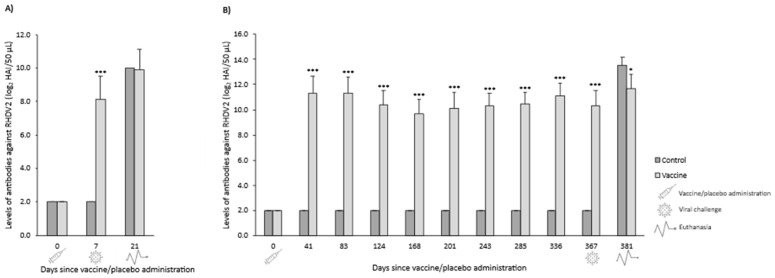
Mean (SD) levels of antibodies against rabbit hemorrhagic disease virus type 2 (RHDV2) in vaccine and control groups of studies evaluating onset of immunity (**A**) and duration of immunity (**B**) against a highly virulent RHDV2 strain. * *p* < 0.05; *** *p* < 0.001 vs. controls.

In both OOI and DOI trials, control rabbits were seronegative for RHDV2 from the day they received the placebo (day 0) until the day they were challenged (days 7 and 367 for OOI and DOI, respectively). In the OOI trial, seven days after administering the vaccine or placebo, the levels of antibodies against RHDV2 were statistically significantly higher (*p* < 0.001) in vaccinated rabbits compared with control rabbits. However, no differences were observed 14 days after being challenged (day 21) (Figure 7A). Similarly, in the DOI trial, vaccinated rabbits exhibited statistically significantly higher (*p* < 0.001) levels of antibodies against RHDV2 compared with control rabbits at all time points assessed after administering the vaccine or placebo, except for the last one (day 381, 14 days after being challenged), when the levels of antibodies of controls increased above those of vaccinated rabbits (Figure 7B).

### 3.3. Protection Against Classical RHDV

#### 3.3.1. Mortality and Clinical Signs

In the OOI trial, 0 (0.0%) rabbits died in the vaccine group and 10 (90.9%) in the control group, with all deaths in the control group occurring between 24 and 48 h after the challenge. The analysis of liver samples showed that all rabbits dying after the challenge presented the classical RHDV in their liver.

In the trial assessing the DOI, one (10.0%) and eight (80.0%) rabbits died in the vaccine and control groups, respectively. All deaths in the control group occurred between 24 and 72 h after the challenge. The analysis of liver samples showed the presence of classical RHDV in all rabbits dying after the challenge.

**Figure 8:**
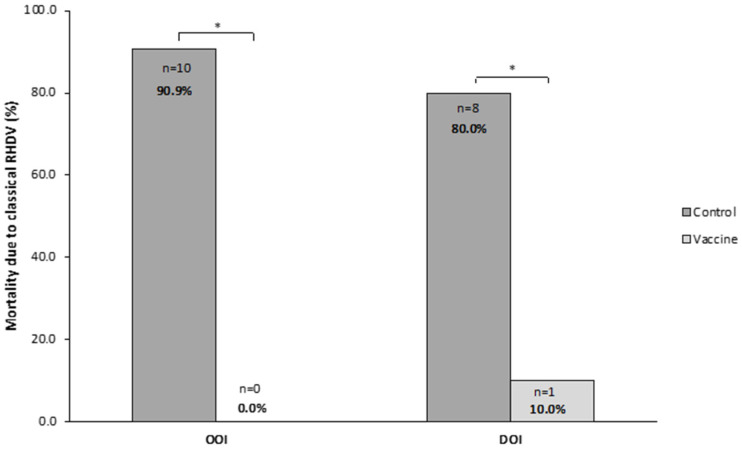
Mortality caused by the classical rabbit hemorrhagic disease virus (RHDV GI.1) in vaccine and control groups of studies evaluating onset of immunity (OOI) and duration of immunity (DOI) against a classical RHDV strain. * *p* < 0.05.

Therefore, both OOI and DOI trials showed that the mortality due to classical RHDV was statistically significantly higher (*p* < 0.05) in rabbits receiving the placebo than in those receiving the vaccine (Figure 8).

As for clinical signs, none of the rabbits from any treatment group showed clinical signs related to RHD within the 14 days after the challenge.

#### 3.3.2. Serological Response

**Figure 9:**
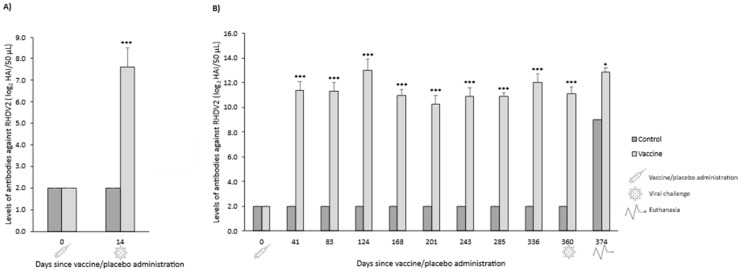
Mean (SD) levels of antibodies against rabbit hemorrhagic disease virus type 2 (RHDV2) in vaccine and control groups of studies evaluating onset of immunity (**A**) and duration of immunity (**B**) against a classical RHDV (GI.1) strain. * *p* < 0.05; *** *p* < 0.001 vs. controls.

In both OOI and DOI trials, control rabbits were seronegative for RHDV2 from the day they received the placebo (day 0) until the day they were challenged (days 14 and 360 for OOI and DOI, respectively). The levels of antibodies against RHDV2 were statistically significantly higher (*p* < 0.05) in vaccinated rabbits compared with control rabbits at all time points assessed after administering the vaccine or placebo (Figure 9).

## 4. Discussion

This study assessed the protective efficacy of a new recombinant vaccine against different RHDV strains (RHDV2, hvRHDV2, and classical RHDV) through OOI and DOI trials. Overall, the results demonstrated protection in vaccinated rabbits against all challenge strains, including the hvRHDV2. Specifically, the results observed indicate that mortality was significantly lower or totally prevented in vaccinated rabbits compared to controls following heterologous challenges. Consistent with these clinical outcomes, the serological analyses showed that vaccinated rabbits developed detectable RHDV2 antibodies as early as 7 days post-vaccination, and these antibody levels were maintained up to twelve months, suggesting a durable and robust immune response.

Previous studies assessing the efficacy of other vaccines against RHDV2 (i.e., vectored, inactivated or multivalent) also showed that rabbits infected with RHDV2 died less frequently if they had previously been vaccinated [15,16,17,18,19], with mortality rates ranging from 0.0% to 20.0%, consistently with those found in this study (0.0% to 12.5%). However, only two of these studies challenged the animals with both RHDV2 and classical RHDV strains, with one of them using a vectored vaccine based on attenuated myxoma virus strains in which VP60 genes of RHDV2 and classical RHDV were inserted separately [16], and the other used a multivalent vaccine consisting of a mix of inactivated, whole viruses (one RHDV2 and two classical RHDV strains) [17]. The latter also tested the efficacy of a monovalent vaccine based on inactivated RHDV2, but it did not show cross-protection against any of the classical RHDV strains [17].

In addition, the RHDV2 strains used in these studies were isolated before the hvRHDV2 strain used in the present study. Thus, to our knowledge, this is the first study to report the efficacy of a vaccine based on a single recombinant RHDV2 protein that shows protection against two RHDV2 pathotypes (one highly virulent strain and one less virulent), as well as against the classical RHDV strain. The protection against recent highly virulent strains has also been confirmed in another study where rabbits were challenged with a hvRHDV2 strain isolated from an RHD outbreak in The Netherlands in 2022 [20], representing a significant advancement in RHD prevention strategies.

While several previous reports have described limited or absent cross-protection of RHDV2-based vaccines against classical RHDV, particularly for monovalent inactivated RHDV2 formulations, the protection observed across distinct RHDV strains suggests that the immune response elicited by the recombinant antigen likely targets conserved epitopes within the VP60 capsid protein. The VP60 antigen used in the vaccine is derived from a single genotype 2 (RHDV2) field strain. While the antigen originates from RHDV2, its conserved domains (S and P1) likely contribute to cross-protective immunity against hvRHDV2 and classical RHDV strains. Indeed, VP60 is known to contain regions of strong sequence conservation between RHDV2 and RHDV, particularly within the S and P1 domains, which may explain the broad neutralizing capacity of antibodies induced by the vaccine [2,9,21]. Although the precise molecular mechanisms of cross-protection remain to be elucidated, structural analyses of *Lagovirus* capsid have shown stable domain architecture (S, P1) and capsomer interactions, which suggest that conserved amino acid motifs may help to preserve conformational epitopes essential for antibody recognition [9,21]. Further immunological and structural studies would be required to define which epitopes and mechanisms underly the broad protective response observed.

In addition, VLPs, such as those formed by the VP60 protein, maintain the native conformation and repetitive antigenic structure of the viral capsid while being non-replicative, allowing for efficient recognition by the immune system [22]. The VLP architecture facilitates B-cell receptor binding and promotes strong humoral immunity, which may account for the high antibody titers and rapid seroconversion observed in vaccinated animals.

Thus, the results presented in this study support the use of recombinant VLP-based vaccines as a technologically advanced alternative to inactivated or multivalent vaccines for RHD control.

## 5. Conclusions

The results of our study indicate that the recombinant vaccine is the first to induce robust and durable protection against RHD caused by the RHDV2, hvRHDV2, and classical RHDV strains.

## 6. Patents

Fontseca M., Nadal G., and Montané J. are inventors of a patent application filed by HIPRA SCIENTIFIC S.L.U. based on some of the results from the present work.

## Figures and Tables

**Table 1:** Main characteristics of each trial.

Virus Tested	Type of Trial	Age of Rabbits at the Time of Vaccine or Placebo Administration	Time of the Virus Challenge	Infective Dose for the Virus Challenge
RHDV2	OOI	30 days	7 days after vaccine or placebo administration	2^15^ HA
RHDV2	DOI	30 days	12 months after vaccine or placebo administration	2^15^ HA
highly virulent RHDV2	OOI	30 days	7 days after vaccine or placebo administration	2^15^ HA
highly virulent RHDV2	DOI	30 days	12 months after vaccine or placebo administration	2^15^ HA
Classical RHDV (GI.1)	OOI	10 weeks	14 days after vaccine or placebo administration	2^12^ HA
Classical RHDV (GI.1)	DOI	30 days	12 months after vaccine or placebo administration	2^12^ HA

DOI, duration of immunity; HA, hemagglutination assay; OOI, onset of immunity; RHDV, rabbit hemorrhagic disease virus; RHDV2, rabbit hemorrhagic disease virus type 2.

## Data Availability

The data presented in this study are available on request from the corresponding author due to intellectual property protection and confidentiality.

## Supplementary Materials

The following supporting information can be downloaded at https://www.mdpi.com/article/10.3390/vaccines14020123/s1. Table S1: Clinical signs and scores recorded during the trials.

## Abbreviations

The following abbreviations are used in this manuscript:

BEI	Binary ethyleneimine
Classical RHDV	Classical rabbit hemorrhagic disease virus GI.1
DOI	Duration of immunity
HAI	Hemagglutination inhibition assay
hvRHDV2	Highly virulent rabbit hemorrhagic disease virus GI.2
OOI	Onset of immunity
RHD	Rabbit hemorrhagic disease
RHDV	Rabbit hemorrhagic disease virus
RHDV2	Rabbit hemorrhagic disease virus GI.2
