# Supplementary material for: Protection Against Rabbit Hemorrhagic Disease Virus Strains: Efficacy of a New Commercial Recombinant RHDV2 Capsid Protein VP60 Vaccine
Source: Vaccines (Basel). 2026 Jan 27;14(2):123. doi: 10.3390/vaccines14020123 (PMC12945178; doi:10.3390/vaccines14020123)
Supplement: Supplementary file 1 [file vaccines-14-00123-s001.zip › vaccines-4073638-supplementary.pdf]

Protection against Rabbit Haemorrhagic Disease Virus Strains: Efficacy of a New Commercial Recombinant RHDV2 Capsid Protein VP60 Vaccine

Supplementary file 1

Tables

Table S1. Clinical signs and scores recorded during the trials.

	Score	Description
**Depression**	0	Active animal. Responsive to stimuli and resistant to immobilization.
	1	Less active but responsive to weak stimuli.
	2	Not responsive to weak but to strong stimuli.
	3	Not responsive to strong stimuli.
**Body condition**	0	Good body condition.
	1	Impaired body condition.
	2	Severe growth delay.
**Dyspnea**	0	Normal
	1	Dyspnea
**Nasal discharge**	0	Normal
	1	Nasal discharge with blood
**Ocular discharge**	0	Normal
	1	Ocular discharge
	2	Ocular hemorrhagic discharge
